# Supplementary material for: An open-source framework for large-scale, flexible evaluation of biomedical text mining systems
Source: J Biomed Discov Collab. 2008 Jan 29;3:1. doi: 10.1186/1747-5333-3-1 (PMC2276192; doi:10.1186/1747-5333-3-1)
Supplement: Additional file 1 — Evaluation platform source code. The additional file contains the software evaluation framework discussed in this paper. The most current release can be downloaded from SourceForge.net [1]. See the accompanying README file for instructions on installation and use. Also included in the distribution is a collection of UIMA wrappers for some commonly used BioNLP tools and annotated corpora. [file 1747-5333-3-1-S1.zip › bionlp_uima_repository-1.0.1/docs/api/src-html/edu/uchsc/ccp/util/external/stanfordparser/TypedDependencyCls.html]

```
001    package edu.uchsc.ccp.util.external.stanfordparser;
002    
003    public class TypedDependencyCls {
004    
005        private int governorTokenNum;
006    
007        private int dependentTokenNum;
008    
009        private String relation;
010    
011        public TypedDependencyCls(int governorToken, int dependentToken, String relation) {
012            this.dependentTokenNum = dependentToken;
013            this.governorTokenNum = governorToken;
014            this.relation = relation;
015        }
016    
017        public int getDependentTokenNum() {
018            return dependentTokenNum;
019        }
020    
021        public void setDependentTokenNum(int dependentTokenNum) {
022            this.dependentTokenNum = dependentTokenNum;
023        }
024    
025        public int getGovernerTokenNum() {
026            return governorTokenNum;
027        }
028    
029        public void setGovernerTokenNum(int governorTokenNum) {
030            this.governorTokenNum = governorTokenNum;
031        }
032    
033        public String getRelation() {
034            return relation;
035        }
036    
037        public void setRelation(String relation) {
038            this.relation = relation;
039        }
040    
041        public String toString() {
042            return "GOV: " + governorTokenNum + "  DEP: " + dependentTokenNum + "  REL: " + relation;
043        }
044        
045       
046        
047    
048    }
```
